# Pediatric differentiated thyroid carcinoma in stage I: risk factor analysis for disease free survival

**DOI:** 10.1186/1471-2407-9-306

**Published:** 2009-09-01

**Authors:** Nobuyuki Wada, Kiminori Sugino, Takashi Mimura, Mitsuji Nagahama, Wataru Kitagawa, Hiroshi Shibuya, Keiko Ohkuwa, Hirotaka Nakayama, Shohei Hirakawa, Yasushi Rino, Munetaka Masuda, Koichi Ito

**Affiliations:** 1 Surgical Branch, Ito Hospital, 4-3-6 Jingumae, Shibuya-ku, Tokyo 150-8308, Japan; 2 Department of Surgery, Yokohama City University Hospital, 3-9 Fukuura, Kanazawa-ku, Yokohama City, Kanagawa 236-0004, Japan

## Abstract

**Background:**

To examine the outcomes and risk factors in pediatric differentiated thyroid carcinoma (DTC) patients who were defined as TNM stage I because some patients develop disease recurrence but treatment strategy for such stage I pediatric patients is still controversial.

**Methods:**

We reviewed 57 consecutive TNM stage I patients (15 years or less) with DTC (46 papillary and 11 follicular) who underwent initial treatment at Ito Hospital between 1962 and 2004 (7 males and 50 females; mean age: 13.1 years; mean follow-up: 17.4 years). Clinicopathological results were evaluated in all patients. Multivariate analysis was performed to reveal the risk factors for disease-free survival (DFS) in these 57 patients.

**Results:**

Extrathyroid extension and clinical lymphadenopathy at diagnosis were found in 7 and 12 patients, respectively. Subtotal/total thyroidectomy was performed in 23 patients, modified neck dissection in 38, and radioactive iodine therapy in 10. Pathological node metastasis was confirmed in 37 patients (64.9%). Fifteen patients (26.3%) exhibited local recurrence and 3 of them also developed metachronous lung metastasis. Ten of these 15 achieved disease-free after further treatments and no patients died of disease. In multivariate analysis, male gender (p = 0.017), advanced tumor (T3, 4a) stage (p = 0.029), and clinical lymphadenopathy (p = 0.006) were risk factors for DFS in stage I pediatric patients.

**Conclusion:**

Male gender, tumor stage, and lymphadenopathy are risk factors for DFS in stage I pediatric DTC patients. Aggressive treatment (total thyroidectomy, node dissection, and RI therapy) is considered appropriate for patients with risk factors, whereas conservative or stepwise approach may be acceptable for other patients.

## Background

Differentiated thyroid carcinoma (DTC) is rare in pediatric patients. The incidence of this form has been reported to range from 2.6% to 12.9% of whole DTC population treated during the same period [1-11]. Papillary thyroid carcinoma (PTC) is the most common thyroid malignancy in pediatric patients as well as in adult patients [1-35]. Radiation exposure in infancy has been reported to be associated with the possible occurrence of PTC [12,13]. Follicular thyroid carcinoma (FTC) is the next common form, but is usually quite rare in pediatric patients. Advanced tumor manifestations, such as large or extensive tumor, multifocality, lymphadenopathy, and synchronous lung metastasis at diagnosis, are more evident and are indicated to be risk factors in pediatric patients [6,14-18]. In addition, the lower age population develops a worse prognosis compared to young adult or adolescent patients [11,15,17,19-22]. Therefore, total or near total thyroidectomy followed by radioactive iodine (RI) therapy is recommended as the optimal treatment strategy [1-7,12,18-20,23-28]. However, others advocate that less aggressive treatment is sufficient and that the extent of surgery is not related to the clinical outcome [8,9,22,29-34]. Additionally, extended surgery increases the potential risk for surgical complications, such as recurrent laryngeal nerve (RLN) injury or permanent hypoparathyroidism [21,23,32]. Thus, because of the clinical discrepancy between aggressive manifestation and better outcomes, controversy still remains concerning the appropriate treatment for pediatric DTC patients. The American Joint Committee on Cancer (AJCC)/International Union Against Cancer (UICC) tumor-node-metastasis (TNM) staging system defines pediatric DTC patients as TNM stage I or stage II according to the presence or absence of synchronous distant metastasis, regardless of the degree of extrathyroid extension or lymph node metastases. In general, stage II patients receive total thyroidectomy followed by RI therapy and their lung metastases well respond to RI treatment [4,11,14,16,19-21,25,27,33,34]. On the other hand, the treatment strategy for stage I pediatric patients remains controversial; a more conservative approach may be suitable for these patients [8,9,22,26,29-34]. In this study we therefore analyzed the risk factors in stage I pediatric DTC patients.

## Methods

### Patient

Between 1962 and 2004, 68 pediatric patients (≤ 15 years) with DTC were initially treated at Ito Hospital, which has a special interest in thyroid disease. Among the 68 consecutive patients, 11 patients were defined as TNM stage II because of the presence of synchronous lung metastasis. The remaining 57 TNM stage I patients (7 males and 50 females) were reviewed in this study. The mean age (mean ± standard deviation [SD]) was 13.1 ± 2.3 (range: 7-15) years. The mean follow-up period was 17.4 ± 10.9 (range: 0.7-45.0) years. In 42 patients (73.7%), the duration of follow-up was over 10 years. Their mean height and body weight were 152.7 ± 12.4 (range: 118.3-172.0) cm and 45.4 ± 12.2 (range: 18.0-73.0) kg, respectively. All 57 patients in our series underwent initial treatment at our institution and none of the patients had undergone any previous treatment for DTC elsewhere. Thus, patients with any prior management before diagnosis and therapy in our institution were excluded from the present analysis.

### Histology

There were 46 PTCs including 2 follicular variant types and 11 FTCs. Overall, the primary tumor size was 4.3 ± 2.0 cm. Specific variants, such as follicular variant of papillary carcinoma, solid and trabecular pattern, squamous metaplasia or marked psammoma bodies, were found in 15 patients. Hürthle cell carcinoma or insular carcinoma was not found in our series. Medullary thyroid carcinoma or anaplastic thyroid carcinoma were excluded.

### Clinical manifestation at diagnosis

The clinical manifestations at diagnosis were thyroid mass in 53 (93.0%), extrathyroid extension in 7 (12.3%), clinical lymphadenopathy in 12 (21.1%), and hoarseness due to RLN palsy in 2 (3.5%) of all 57 patients. No patients presented with synchronous lung metastasis at diagnosis but three patients developed metachronous lung metastasis in the follow up period. In only one patient, PTC was incidentally found by ultrasound examination due to screening during the treatment for Graves' disease.

### Family or radiation history

Eleven patients had a family history of thyroid disease (PTC in one, benign thyroid tumor such as adenomatous goiter or follicular adenoma in 6, and Graves's disease in 4). There were no patients who had previous radiation exposure to the head and neck.

### Preoperative diagnostic modality

Fine needle aspiration biopsy cytology (FNABC) has been performed since 1974 and ultrasound (US) examination has been performed as a routine preoperative practice to study primary thyroid tumor and regional lymph nodes since 1980 at Ito Hospital. US-guided FNABC has also been performed as a novel diagnostic tool since 1998. Overall, FNABC was performed preoperatively in 36 patients. Cytological diagnoses were PTC, strongly suspected PTC, suspected FTC, and follicular adenoma in 25, 2, 3, and 6 of the 36 patients, respectively. Consequently, conventional or US-guided FNABC contributed to the diagnosis of thyroid carcinoma in 30 of 36 (83.3%) patients before surgery.

Chest X-ray was preoperatively performed as one of the routine examinations for total anesthesia and as a screening purpose of apparent lung metastasis in all patients. Computed tomography (CT) scan was also performed before surgery to evaluate local disease and the presence or absence of synchronous lung metastasis since 1987.

The level of serum thyroglobulin (Tg) was measured since 1981 and the presence of Tg antibody was evaluated since 1994. In 7 patients, the information of serum Tg was not available. Finally, the level of serum Tg was considered reliable in 15 patients without Tg antibody at the end of follow-up. Thus, the serum Tg was valuable to diagnose disease recurrence or progression only in selected patients in our series. Therefore, the level of serum Tg was not evaluated statistically in this study.

### Surgical treatment

Lobectomy and subtotal or total thyroidectomy were performed in 25 and 21 of 46 PTC patients, and in 9 and 2 of 11 FTC patients, respectively. Of the 46 PTC patients, 10, 1, 31, and 4 patients underwent no dissection, only central node dissection, ipsilateral modified neck dissection (MND), and bilateral MND, respectively. Of the 11 FTC patients, 6, 2, and 3 patients underwent no dissection, only central node dissection, and ipsilateral MND, respectively. MND was defined as systematic node dissection in the central compartment (level VI) and ipsilateral lateral compartment (level II to V). Fundamentally, lobectomy was performed in patients with unifocal and unilateral tumor, no clinical lymphadenopathy, and no distant metastasis at diagnosis. Total thyroidectomy was performed in patients with multifocal or invasive tumor, clinical lymphadenopathy, or distant metastasis at diagnosis. Subtotal thyroidectomy was performed to avoid surgical complications, such as permanent hypoparathyroidism or RLN palsy, in selected patients who were basically candidates for total thyroidectomy. For example, patients presenting with ipsilateral RLN palsy underwent less total thyroidectomy to prevent bilateral RLN palsy. MND was usually performed in patients with advanced tumor stage or lymphadenopathy, whereas no or only central node dissection was performed in those with lower tumor stage or no lymphadenopathy.

### Pathological diagnosis and TNM staging

Primary tumors and regional lymph nodes were removed in all patients and those specimens were pathologically confirmed to be PTCs or FTCs and nodal metastases.

The sixth edition of the AJCC/UICC TNM classification was used to determine the tumor (T) stage, nodal (N) stage, distant metastasis (M) stage, and TNM staging. Among the 68 pediatric patients, 57 patients without synchronous distant metastasis were defined as TNM stage I and were analyzed statistically in this study. Eleven patients with synchronous lung metastasis were defined as TNM stage II and were excluded from the present analysis.

### Radiotherapy

Therapeutic RI treatment has principally been performed when refractory nodal recurrence was observed or when distant metastasis was detected visually by chest X-ray or CT scan. Overall, 6 patients underwent RI ablation and 4 patients received therapeutic dose of RI because 3 patients developed both nodal recurrence and metachronous lung metastasis and one patient developed nodal recurrence.

External beam radiation therapy (EBRT) was performed for only one patient.

### TSH suppression therapy

TSH suppression therapy was administered to all patients but it was adequately maintained in 48 of 57 patients. In the remaining 9 patients, TSH suppression was inadequate due to patient compliance, or was not maintained because of the curability of the disease (unilateral tumor and no pathological node metastasis). These 9 patients did not develop any recurrent disease at the time of the present analysis.

### Therapeutic complications

Overall, permanent surgical complications considered iatrogenic were found in 9 (15.8%) of 57 patients. Permanent hypoparathyroidism was observed in 6 (10.5%) patients (subtotal thyroidectomy in one and total thyroidectomy in 5). RLN palsy considered due to the nerve injury not due to the potential risk of primary disease was observed in 3 (5.3%) patients (subtotal thyroidectomy in one and total thyroidectomy in 2). None of the 10 patients (3 males and 7 females) who received RI ablation or therapy developed any other secondary malignant disease. Among the 7 female patients, 2 patients experienced 3 healthy deliveries.

### Follow-up and definition of disease recurrence

Patient follow-up was generally performed every 6 months after a relatively stable condition was obtained. Recurrent disease was defined as local recurrence (remnant thyroid tissue or regional lymph node) or metachronous distant metastasis that developed as new disease during the follow-up period, at least 6 months after the initial surgery. Recurrence was detected by physical and radiological examinations, including US, chest X-ray, CT scan, and/or scintigraphy, and blood test to screen for elevation of the serum Tg in patients treated with total thyroidectomy.

### Evaluation of outcomes

Clinicopathological features, treatments, and outcomes, such as age stratification (≤ 13 years old), male gender, histology (papillary carcinoma), specific variant, tumor size (>3.0 cm), extrathyroid extension, multifocality, tumor stage (T3, 4a), clinical lymphadenopathy (cN), pathological node metastasis (pN), the extent of thyroid resection and node dissection, surgical complications, and the rates of disease recurrence and mortality were evaluated. These factors were compared between patients who developed recurrence and those who did not. Disease-free survival (DFS) was evaluated in all and univariate and multivariate analyses were then performed to reveal risk factors for DFS. Disease-specific survival was not evaluated because no patients died of disease. This study was approved by the Ethical boards of our institution. Informed consent was obtained from patients and their parents.

### Statistical analysis

The results are expressed as the mean ± standard deviation (SD). Frequencies were compared with the chi-square test and Fisher's exact probability test. DFS was assessed with the Kaplan-Meier method and compared with the log-rank test. The effects of each risk factor on DFS were evaluated by multivariate analysis using the Cox proportional hazards models [Hazard ratio (HR), 95% confidence interval (CI)]. Differences were considered statistically significant when p-values were less than 0.05. Statistical analyses were performed with SPSS, version 11.0.

## Results

### Clinicopathological features and outcomes

Table 1 summarizes the clinicopathological features and outcomes. 26.3% (15 patients) exhibited recurrent disease (local in 12 and both local and lung metastasis in 3). In the 15 patients (regional node recurrence in 13 and both remnant thyroid and regional node recurrence in 2), the clearance of local recurrences was achieved successfully. After the treatment for recurrence, 10 of the 15 patients are currently free of disease. Four patients with nodal recurrence and one patient with both nodal recurrence and lung metastasis are alive with disease.

**Table 1:** Clinicopathological features in 57 TNM stage I pediatric DTC patients

			Recurrence				
			
Clinicopathological features	Total (n = 57)		Yes (n = 15)		No (n = 42)		P-value
	No.	%	No.	%	No.	%	
Age (≤13)	23	40.4	8	53.3	15	35.7	NS
Male gender	7	12.3	5	33.3	2	4.8	0.011
Histology (papillary carcinoma)	46	80.7	15	100.0	31	73.8	NS
Specific variant	15	26.3	4	26.7	11	26.2	NS
Tumor size (>3.0 cm)	38	66.7	10	66.7	28	66.7	NS
Extrathyroid extension	7	12.3	3	20.0	4	9.5	NS
Multifocality	18	31.6	9	60.0	9	21.4	0.010
Tumor stage (T3, T4a)	30	52.6	10	66.7	20	47.6	NS
Clinical lymphadenopathy (cN)	12	21.1	7	46.7	5	11.9	0.009
Pathological node metastasis (pN)	37	64.9	13	86.7	24	57.1	0.040
Subtotal/total thyroidectomy	23	40.4	8	53.3	15	35.7	NS
Modified neck dissection	38	66.7	13	86.7	25	59.5	NS

NS: not significance

### Risk factor analysis

As shown in Table 1, male gender (p = 0.011), multifocality (p = 0.010), cN (p = 0.009), and pN (p = 0.040) differed significantly between patients who developed recurrence and those who did not.

**Figure 1:**
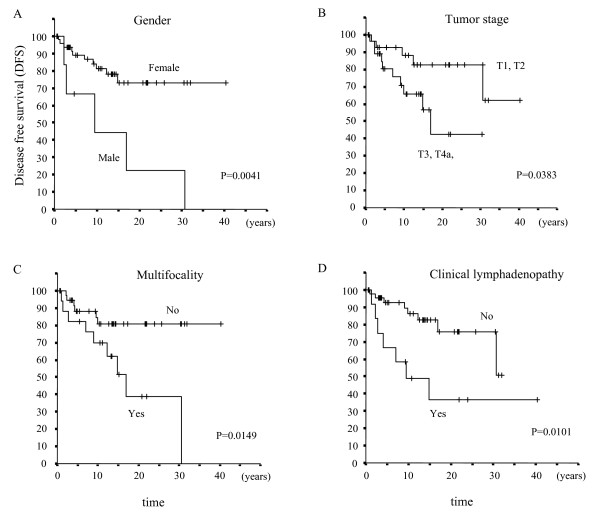
**Kaplan-Meier survival curves for disease-free survival (DFS) in 57 TNM stage I pediatric DTC patients according to risk factors, gender (A), tumor stage (B), multifocality (C), and clinical lymphadenopathy (D)**.

DFS Kaplan-Meier curves in 57 stage I patients are shown in Figure 1. DFS curves significantly differed according to gender (Fig. 1A), tumor stage (Fig. 1B), multifocality (Fig. 1C), and cN (Fig. 1D). These risk factors showed statistical significance by univariate analysis. In multivariate analysis, male gender (p = 0.017, HR 4.51, 95%CI: 1.31-15.5), tumor stage (p = 0.029, HR 1.06, 95%CI: 1.01-1.12), and cN (p = 0.006, HR 4.77, 95%CI: 1.55-14.7) were significant risk factors for DFS in stage I patients, as shown in Table 2.

**Table 2:** Risk factor analysis for disease free survival (DFS) in 57 TNM stage I pediatric DTC patients.

	Univariate			Multivariate		
	
	HR	95%CI	P-value	HR	95%CI	P-value
Male gender	4.36	1.46-13.0	0.008	4.51	1.31-15.5	0.017
Tumor stage (T3, T4a)	1.06	1.00-1.11	0.050	1.06	1.01-1.12	0.029
Multifocality	3.36	1.19-9.43	0.022	2.14	0.71-6.41	0.175
Clinical lymphadenopathy	3.51	1.27-9.71	0.016	4.77	1.55-14.7	0.006

HR: hazard ratio. CI: confidence interval.

## Discussion

Demographically, the incidence of pediatric DTC patients ranged from 2.6% to 12.9% of the whole DTC population [1-11]. PTC is the most common histological type of thyroid carcinoma in pediatric patients, whereas FTC is uncommonly found [1-35]. In our series, PTCs and FTCs were found in 46 patients (80.7%) and in 11 patients (19.3%), respectively. Moreover, none of these 11 FTCs presented with distant metastasis both at diagnosis and during the follow-up period and they developed a better prognosis than PTCs. This was in concordance with the results of previous studies [14,28]. Radiation exposure is considered to be etiologically associated with the occurrence of PTC and the incidence outside of the Belarus region usually ranges from 0% to 20% [1-7,11,12,15-17,19-28,33,35]. Spinelli et al. [12] and Viswathan et al. [13] suggested that radiation-induced thyroid carcinomas are at risk for worse prognosis.

Physical examination is important because thyroid mass and lymphadenopathy are more common in pediatric DTC patients. Thyroid mass is found in 31% to 97% of the pediatric patients [2-8,10,11,13,16-18,22-27,35] and clinical lymphadenopathy in 12% to 100% [1-8,10,11,13-20,22-27,29-31,34,35]. However, these manifestations are occasionally noticed by parents or medical experts because they develop asymptomatically in this age population, resulting in the difficulty of early diagnosis in pediatric patients. In our series, the thyroid mass was large (mean: 4.3 cm) and was found in 93.0% of the patients. Clinical lymphadenopathy was found in 21.1% of the patients.

Aggressive manifestations at diagnosis are considered the feature of pediatric patients. In the previous studies, extrathyroid extension or advanced tumor stage has been reported to be found in 2.8% to 71% of the pediatric patients [1-9,12-17,21-28,30,31,33,35], multifocality in 12% to 69% [5,7,12-17,22,24,26,28,31,32], pathological node metastasis in 32% to 100% [1-14,17,19-28,30-33,35], and synchronous distant metastasis in 0% to 86% [1-31,33-35]. Our stage I patients presented with extrathyroid extension in 12.3%, advanced tumor stage (T3, T4a) in 52.6%, multifocality in 31.6%, and pathological node metastasis in 64.9%. Some authors have suggested that lung metastasis is significant for disease mortality and the occurrence of lung metastasis is frequent in patients with lymph node metastasis [18,20]. Our findings suggest that advanced tumor stage and clinical lymphadenopathy are independent risk factors for DFS in stage I patients. Borson-Chazot et al. [15] suggest that limited surgery is appropriate for pediatric patients with no lymphadenopathy.

The possible treatments are the main issue to be debated. Total thyroidectomy followed by RI therapy has been recommended as the optimal treatment strategy. However, the occurrence of surgical complication is associated with total thyroidectomy, especially in pediatric patients. In the literature, total thyroidectomy (20% to 100%) has commonly been performed in many centers [1-26,28-35]. Permanent hypoparathyroidism (0% to 35.7%) and RLN palsy (0% to 41%) are the major complications that are usually associated with aggressive surgery [1-7,9,11-13,17,19,21-26,29-33]. Some authors advocate that total thyroidectomy by experts is safe and does not increase the possibility of serious complications [24]. On the other hand, a conservative or stepwise approach has been recommended to prevent surgical complications and to avoid over-surgery for non-advanced disease in pediatric DTC patients [8,9,22,26,29-34]. Our results suggest that stage I pediatric patients with no risk factor are considered candidate for conservative or stepwise treatment.

The clinical discrepancy between advanced tumor manifestations or the high rates of recurrent disease and the low rates of disease mortality has been discussed. In the literature, recurrences occurred in 0% to 47% of the patients [2-13,15-17,19,21-33,35]. However, the rates of disease mortality range from 0% to 5.3% and usually less than 3.0% in the majority of the reports [1-35]. Thus, even patients with advanced disease can achieve a better prognosis after treatment. Brink et al. suggest that stage II pediatric patients can be treated with a stepwise approach and achieve long-term survival [33]. Vassilopoulou-Sellin et al. also suggest that stage II patients have excellent prognosis because of the high concentration of RI [20]. Samuel et al. suggest that complete remission of lung metastases after RI therapy is difficult in some patients, but they usually have no further disease progression and their mortality rate is low [34].

Optimal treatment strategy and significant risk factors for stage I pediatric DTC patients are not well established. Therefore, we analyzed the risk factors in TNM stage I patients. Total thyroidectomy, node dissection, RI therapy, and TSH suppression therapy are considered superior treatment options for pediatric DTC patients in many centers. However, less aggressive treatments are also recommended in other centers. Newman et al. [21] suggested that the selection of the extent of thyroidectomy is warranted, i.e., lobectomy for localized tumor and subtotal or total thyroidectomy for extensive tumor. We consider that the appropriate treatment strategy should be selected to handle primary or recurrent disease and to avoid surgical complications according to risk factors. Such considerations would improve the DFS and the patient's quality of life in pediatric age population. Our results suggest that male gender, advanced tumor stage, and clinical lymphadenopathy are risk factors for DFS in TNM stage I pediatric DTC patients. In the literature, advanced tumor stage, multifocality, lymphadenopathy, distant metastasis, and lower patient age are considered major risk factors for worse prognosis [6,11,14-17,20,21]. Male gender is also a risk factor in pediatric patients [2,11,14]. Thus, TNM stage I pediatric patients may be categorized separately into different substage groups according to risk factors, for example stage IA or stage IB, and a conservative or stepwise approach may be acceptable in pediatric patients without risk factors.

Finally, long-term follow-up is considered essential to evaluate outcomes in pediatric patients [6,35]. A shorter follow-up period may result in inappropriate decision making of the treatment strategy. In the literature, the durations of follow-up period range from 3.8 to 27.6 years [1-6,9-29,31-35]. The mean follow-up period in our study was 17.4 years, which is considered sufficient for evaluating outcomes in pediatric patients.

## Conclusion

Male gender, advanced tumor stage, and clinical lymphadenopathy are risk factors for DFS in TNM stage I pediatric DTC patients, although many patients with recurrence can achieve disease-free after additional treatments. Aggressive treatment (total thyroidectomy with node dissection followed by RI therapy) is appropriate for stage I pediatric patients with risk factors, whereas conservative approach may be acceptable for those without risk factors.

## Competing interests

The authors declare that they have no competing interests.

## Authors' contributions

NW conceived of the study and drafted the manuscript. KS and TM carried out operation of the patients and participated in acquisition of informed consent and study design. MN, WK, HS, KO, HN and SH participated in collection of data. NW, KS, TM and KI participated in acquisition of recurrence data in study subjects and coordinated the study. YR and MM participated in the development of manuscript. All authors have read and approved the final manuscript.

## Pre-publication history

The pre-publication history for this paper can be accessed here:

http://www.biomedcentral.com/1471-2407/9/306/prepub
